# Metabolite and lipid profiling of human vitreous reveals purine metabolic dysregulation and inflammatory lipid remodeling as the drivers of bacterial endophthalmitis

**DOI:** 10.1128/spectrum.00788-26

**Published:** 2026-09-03

**Authors:** Dhanwini Rudraprasad, Agimanailiu Khapuinamai, Sreyasi Maiti, Satyashree Gagan, Joveeta Joseph

**Affiliations:** 1 Jhaveri Microbiology Centre, Brien Holden Eye Research Centre, LV Prasad Eye Institute 28592, Hyderabad, Telangana, India; 2 Manipal Academy of Higher Education 76793, Manipal, Karnataka, India; National Institute of Pharmaceutical Education and Research Hyderabad, Hyderabad, Telangana, India

**Keywords:** vitreous, bacterial endophthalmitis, metabolomics, lipidomics, infection

## Abstract

Bacterial endophthalmitis, an intraocular infection and inflammation, often progresses rapidly and leads to irreversible vision loss, especially in culture-negative cases where the diagnosis is delayed. We profiled infection-associated metabolomic and lipidomic alterations in the vitreous of patients with microbiological and clinically confirmed bacterial endophthalmitis to understand pathogenesis and identify distinct markers that drive infection and retinal injury. Untargeted metabolomic and lipidomic profiling of vitreous samples from affected patients and non-infectious retinal controls was performed using liquid chromatography coupled to tandem mass spectrometry. Metabolites and lipids with *P*-value < 0.05 were considered for further pathway-specific analysis. Metabolomic profiling distinctly segregated infected from control samples, revealing dysregulation in purine metabolism, amino acid turnover, polyamine synthesis, redox regulation, and vitamin pathways. Key metabolites, xanthine, hypoxanthine, spermine, seryl-valine, seryl-isoleucine, thymine, O-methyltyramine, and phenyltrimethylammonium, were significantly elevated. These elevations were accompanied by enhanced nucleotide degradation and increased proteolytic activity. Markers of immune activation and oxidative stress were concurrently upregulated, reflecting the broader biochemical disruption characteristic of bacterial endophthalmitis. Antioxidant seleno-L-methionine was downregulated, indicating redox imbalance. Purine, glutathione, vitamin B6, beta-alanine, and arginine-proline metabolism were broadly disrupted. Complementary lipidomics showed extensive vitreous remodeling. Alongside, fatty acids, bile acid derivatives, and membrane lipids were also dysregulated. Bacterial endophthalmitis triggers strong and consistent changes in metabolite and lipid profiles within vitreous, which reflect alterations in immune activation, oxidative stress, tissue breakdown, and metabolic reprogramming. The persistence of these biomolecular signatures in the vitreous highlights host response to infection and subsequent mechanistic elucidation of the specific drivers of retinal inflammation and progressive tissue damage.

**IMPORTANCE:**

The study highlights that bacterial endophthalmitis is not only driven by microbial burden but also by host metabolic and lipidomic reprogramming, transforming the understanding of disease pathogenesis. This study reveals metabolite–lipid networks in ocular infections, laying a critical foundation for precision medicine strategies in endophthalmitis management. Specifically, we found significant increases in key metabolites including xanthine, hypoxanthine, spermine, seryl-valine, seryl-isoleucine, thymine, O-methyltyramine, and phenyltrimethylammonium, together reflecting increased nucleotide degradation, proteolytic activity, and immune metabolic reprogramming. These molecular signatures not only provide deeper mechanistic insights into ocular infection but also provide potential biomarkers that could aid in diagnosis and monitoring of infection when conventional microbiological methods are inconclusive. The results highlight the clinical importance of metabolic profiling as a powerful tool to optimize visual outcomes, guide adjunctive therapeutic decisions, and enhance prognostic evaluation in patients affected by the condition.

## INTRODUCTION

Endophthalmitis is a severe intraocular inflammatory condition that can progress rapidly to irreversible blindness even after prompt diagnosis and treatment (1). The disease is caused by a broad spectrum of bacterial pathogens, with their prevalence varying based on the geographic region and clinical context. Gram-positive cocci remain the predominant etiologic group worldwide, including *Staphylococcus aureus*, *Staphylococcus epidermidis*, *Staphylococcus lugdunensis*, *Staphylococcus gallinarum*, *Staphylococcus kloosii*, *Streptococcus mitis*, and *Enterococcus faecalis* (2). *Bacillus cereus* and *Bacillus licheniformis* represent significant etiologic agents, especially in post-traumatic endophthalmitis (3). Gram-negative organisms such as *Pseudomonas aeruginosa*, *Pseudomonas fluorescens*, *Pseudomonas stutzeri*, and *Achromobacter xylosoxidans* are importantly increasing in cases of endophthalmitis (4). Notably, many of these isolates, particularly *Pseudomonas* and *Staphylococcus* species, are linked to an aggressive disease trajectory, swift intraocular devastation, and suboptimal visual prognosis if therapeutic intervention is postponed (5).

The retina is a highly specialized and metabolically active neural tissue, exhibiting marked vulnerability to inflammatory damage (6). During infectious endophthalmitis, dysregulated immune activation, oxidative stress, and the release of cytokines such as IL-6, TNF-α, and IL-1β contribute to blood–retinal barrier breakdown, vascular leakage, edema, and progressive retinal degeneration (7, 8). A major challenge in clinical management is the rapid increase of culture-negative cases, which limits definitive pathogen identification and delays targeted therapy. Previous data from our institute demonstrated that more than 70% of endophthalmitis samples were culture-negative despite clear clinical evidence of infection (9).

Over recent years, metabolomics and lipidomics have emerged as powerful analytical platforms for comprehensively characterizing the global biochemical perturbations that accompany infection-driven inflammatory responses (10). Host immune responses in bacterial endophthalmitis trigger extensive metabolic reprogramming, essential for antimicrobial activity, cytokine secretion, and leukocyte infiltration (11). These dynamic immunological processes profoundly reshape metabolic and lipid signaling networks, particularly those governing lipid biosynthesis, amino acid catabolism, and the production of eicosanoids and other pro-inflammatory mediators (12, 13). Metabolomics and lipidomics profiles within the vitreous, therefore, provide a direct readout of host–pathogen interactions, which could potentially aid in distinguishing infectious endophthalmitis (14). Importantly, such biochemical signatures may remain detectable even when cultures are negative, highlighting these techniques as highly promising diagnostic and mechanistic platforms in endophthalmitis. In ocular infections such as infectious keratitis and conjunctivitis, metabolomics has revealed pathogen-driven disturbances in energy metabolism, oxidative stress, and immune signaling, reflecting both microbial exploitation of host resources and host defense activation (15, 16). Dysregulation of purine metabolism has been demonstrated in bacterial keratitis, while viral keratitis studies have identified metabolite- and extracellular vesicle-based metabolic signatures with high diagnostic accuracy (17). Similarly, in non-infectious ocular surface inflammation, including allergic conjunctivitis, superior limbic keratoconjunctivitis, ocular mucous membrane pemphigoid, and dry eye disease, metabolomic and lipidomic profiling has uncovered characteristic alterations in lipid mediators, amino acid pathways, osmoprotection, oxidative stress, and gut–eye axis metabolites, providing insight into disease mechanisms and biomarker discovery (16).

Comparable trends are reported in posterior-segment disease. In age-related macular degeneration, glaucoma, and diabetic retinopathy, ocular and systemic metabolomic studies consistently demonstrate disease-specific perturbations in amino acid, lipid, redox, mitochondrial, and bioenergetic pathways, highlighting metabolomics as a sensitive readout of retinal stress and inflammation (18). An untargeted multi-omics study by Singh et al. identified upregulation of immune-responsive gene-1 and accumulation of its downstream metabolite itaconate in *Staphylococcus aureus* endophthalmitis in mice, illustrating the central role of immune-metabolic regulation in intraocular infection (19). However, comparable human vitreous metabolomic data are scarce.

In this study, we performed untargeted metabolomic analysis of undiluted vitreous obtained from patients with microbiologically confirmed bacterial endophthalmitis and from non-infectious controls. Vitreous samples were collected at the time of surgery, and inclusion into the study group was based on direct microscopic evidence of bacterial infection following microbiological assessment. Our aim was to characterize infection-associated metabolic alterations within the vitreous microenvironment and to identify potential biochemical signatures that may provide mechanistic insight and support the development of culture-independent diagnostic biomarkers in bacterial endophthalmitis.

## MATERIALS AND METHODS

### Study design

Undiluted vitreous samples were collected from 31 eyes of 31 subjects who underwent pars plana vitrectomy for the diagnosis and/or management of presumed infectious endophthalmitis at the LV Prasad Eye Institute between March 2022 and March 2023. The study cohort included patients ranging in age from 2 to 74 years. All participants underwent comprehensive ophthalmic evaluation at the time of diagnosis and at 6-month follow-up, including slit-lamp biomicroscopy, B-scan ultrasonography, and best-corrected visual acuity assessment. Only cases with well-characterized clinical features of infection and confirmed microbiological diagnosis were included for analysis. Patients with uncertain diagnosis, underlying autoimmune disease, systemic hypertension, or prior steroid use were excluded. The study group comprised 16 patients with microbiologically confirmed bacterial endophthalmitis, including 11 gram-positive and 5 gram-negative infections. The control group consisted of 15 patients undergoing pars plana vitrectomy for non-infectious retinal conditions, including retinal detachment, macular hole, and vitreous hemorrhage, during the same study period.

### Collection and pre-processing of the vitreous humor samples

Vitreous humor (500–1,000 µL) aspirated from both control and infected eyes was processed in accordance with institutional clinical protocols (20, 21). Samples were obtained from the anterior vitreous during pars plana vitrectomy. Approximately 300 µL was aliquoted into a sterile microcentrifuge tube, snap-frozen in liquid nitrogen within 30 min of collection, and stored at −80°C until metabolomic analysis by high-resolution mass spectrometry. The remaining vitreous volume was used for microbiological evaluation. Samples were subjected to direct microscopic examination and cultured on standard bacterial media, including blood agar, chocolate agar, brain heart infusion broth, and thioglycolate medium, as well as fungal media comprising Sabouraud dextrose agar and potato dextrose agar. Bacterial cultures were incubated at 37°C and fungal cultures at 25°C for up to 7 days, following established institutional microbiological protocols.

### Untargeted metabolomics and lipidomics of vitreous humor

#### Sample preparation

Vitreous samples stored at −80°C were thawed on ice (4°C) prior to processing. For combined metabolite and lipid extraction, 150 µL of vitreous humor was mixed with 600 µL of extraction solvent consisting of a 50:50 (vol/vol) ethanol:methanol mixture containing epicatechin (100 ng/mL) as an internal standard. Samples were vortexed, incubated on ice, and agitated in an incubator shaker at 100 rpm for 15 min. Following centrifugation at 10,000 × *g* for 5 min, 600 µL of the supernatant was transferred to fresh tubes and vacuum-dried at room temperature. The dried extracts were reconstituted in 100 µL of methanol and stored on ice for immediate analysis or at −20°C if analysis was delayed. Prior to LC–MS analysis, samples were further reconstituted with 60 µL of solvent, briefly sonicated, and transferred to high-performance liquid chromatography vials with inserts.

### Liquid chromatography–tandem mass spectrometry conditions

Untargeted metabolomic and lipidomic profiling was performed using reverse-phase liquid chromatography coupled to tandem mass spectrometry (LC–MS/MS). Chromatographic separation was achieved on a Zorbax SB-Aq Rapid Resolution High Definition column (3.0 × 150 mm, 5.0 µm, Agilent Technologies, Santa Clara, CA, USA). Mobile phase A consisted of ultra-pure water (18.2 MΩ) with 0.2% acetic acid, and mobile phase B comprised LC–MS–grade methanol containing 0.2% acetic acid. Gradient elution was carried out using a Shimadzu CMB-20A system controller, beginning at 5% B for 2 min, increasing to 35% B over 15 min, followed by a ramp to 95% B over 5 min. The gradient was held at 95% B until 25 min, returned to initial conditions at 25.1 min, and equilibrated until 32 min. The flow rate was maintained at 0.5 mL/min, with autosampler and column oven temperatures set at 15°C and 40°C, respectively.

The LC eluent was introduced into the DuoSpray ion source of a TripleTOF 5600+ mass spectrometer (Sciex, Framingham, MA, USA). Data were acquired in both positive and negative electrospray ionization modes over a mass range of m/z 50–1,000, enabling detection of a broad spectrum of polar metabolites and lipid species. The ion source temperature was maintained at 550°C. High-resolution MS and MS/MS spectra were collected using an information-dependent acquisition method with a collision energy of 35.

### Data acquisition and metabolite/lipid annotation

Raw mass spectrometry (MS) data were acquired using Sciex TF 1.6 software. Feature detection and compound identification were performed using Sciex OS software by matching acquired tandem MS (MS/MS) spectra against the National Institute of Standards and Technology spectral library. Identified metabolites were annotated using the Human Metabolome Database. Lipid and lipid-like molecules were classified based on the chemical ontology using the ClassyFire tool.

### Data pre-processing and normalization

The peak intensity tables were generated from positive and negative ionization modes for metabolomic and lipidomic analysis using MetaboAnalyst 6.0. A sequential quality filtering was applied to the merged data set. Finally, the data were median-normalized and log10 transformed.

### Statistical analysis

Differential metabolite and lipid analysis was performed using two-group *t*-tests and was considered significantly altered only if the adjusted *P*-value < 0.05 and fold change >2. A supervised logistic regression classification framework was implemented using the normalized metabolite intensities. To avoid information leakage and overfitting, feature selection was performed independently within each leave-one-out cross-validation (LOOCV) training fold. In each iteration, one sample was excluded as the test sample, differential metabolite selection was repeated using only the remaining training samples, and the classifier was trained exclusively on the selected metabolites from the training set. The trained model was then used to predict the left-out sample. This procedure was repeated iteratively until every sample had been used once as the independent test sample.

### Pathway analysis

Metabolic and lipid pathway enrichment analysis was conducted using MetaboAnalyst with *Homo sapiens* pathways referenced from the KEGG database. Pathway interaction networks were visualized to identify coordinated metabolic and lipidomic alterations associated with bacterial endophthalmitis. Log-transformed and normalized metabolite and lipid intensity values were exported and processed in Python using NumPy and SciPy. *Z*-score scaling was applied prior to visualization. Clustered heatmaps were generated using the seaborn visualization library, applying hierarchical clustering across both features and samples.

## RESULTS

### Demographic details of the patients included in the study

Among 31 patients included in the study, 16 patients were diagnosed clinically and microbiologically with bacterial endophthalmitis. Additionally, vitreous from 15 non-infectious patients was included as controls (summarized in Table 1). The mean age of patients with endophthalmitis was 45.46 ± 17.43 years, and the control group was 44.0 ± 21.6 years. Severe visual impairment was predominantly observed in the endophthalmitis group with visual acuity worse than 20/200 in 93.7% (15/16) of the patients, whereas the majority of the control patients (60%) had visual acuity between >20/20 and 20/200. Male predominated in both groups, accounting for 81.2% of the endophthalmitis cohort and 66.6% of the control cohort.

**TABLE 1:** Demographic details of patients involved in the study (*N* = 31)^*a*^

Demographics	Endophthalmitis (*n* = 16)	Non-infectious (*n* = 15)
Age (y), mean ± SD	45.46 (±17.43)	44.0 (±21.6)
Visual acuity, *n*%		
<20/200	15 (93.7)	5 (33.3)
>20/20–20/200	1 (6.2)	9 (60)
20/20	–	1 (6.2)
Gender (male; female), *n* (%)	13 (81.2); 3 (18)	10 (66.6); 5 (33.3)
Organisms, *n* (%)		
Gram-positive (68.7)	*Streptococcus mitis* (2), *Staphylococcus gallinarum*, *Enterococcus faecalis*, *Bacillus licheniformis*, *Staphylococcus lugdunensis*, *Staphylococcus kloosii*, *Bacillus cereus* (3), and *Staphylococcus epidermidis*	–
Gram-negative (31.2)	*Pseudomonas fluorescens*, *Achromobacter xylosoxidans*, *Pseudomonas stutzeri* (2), and *Pseudomonas aeruginosa*	–
Diagnosis, *n* (%)		
Traumatic	11 (68.7)	–
Postoperative	5 (31.2)	–
Retinal detachment	–	7 (46.6)
Macular hole	–	3 (20)
Epiretinal membrane	–	2 (13.3)
Retinitis	–	1 (6.6)
Retinochoroiditis	–	1 (6.6)
Retinal vasculitis	–	1 (6.6)

^*a*^ “–” indicates that no patients were recruited under the respective category or section.

Microbiological analysis revealed that gram-positive organisms accounted for 68.7% of the total endophthalmitis cases, which included *Streptococcus mitis* (*n* = 2), *Staphylococcus gallinarum*, *Enterococcus faecalis*, *Bacillus licheniformis*, *Staphylococcus lugdunensis*, *Staphylococcus kloosii*, *Bacillus cereus* (*n* = 3), and *Staphylococcus epidermidis*. Gram-negative organisms accounted for 31.2% of cases and included *Pseudomonas fluorescens*, *Achromobacter xylosoxidans*, *Pseudomonas stutzeri* (*n* = 2), and *Pseudomonas aeruginosa*. Traumatic etiology was the most common (68.7%), followed by postoperative endophthalmitis in 31.2%. The control group comprises patients with non-infectious retinal conditions such as retinal detachment (46.6%), macular hole (20%), epiretinal membrane (13.3%), retinitis (6.6%), retinochoroiditis (6.6%), and retinal vasculitis (6.6%).

### Global metabolomic analysis demonstrated distinct separation between control and bacterial endophthalmitis vitreous

**Fig 1:**
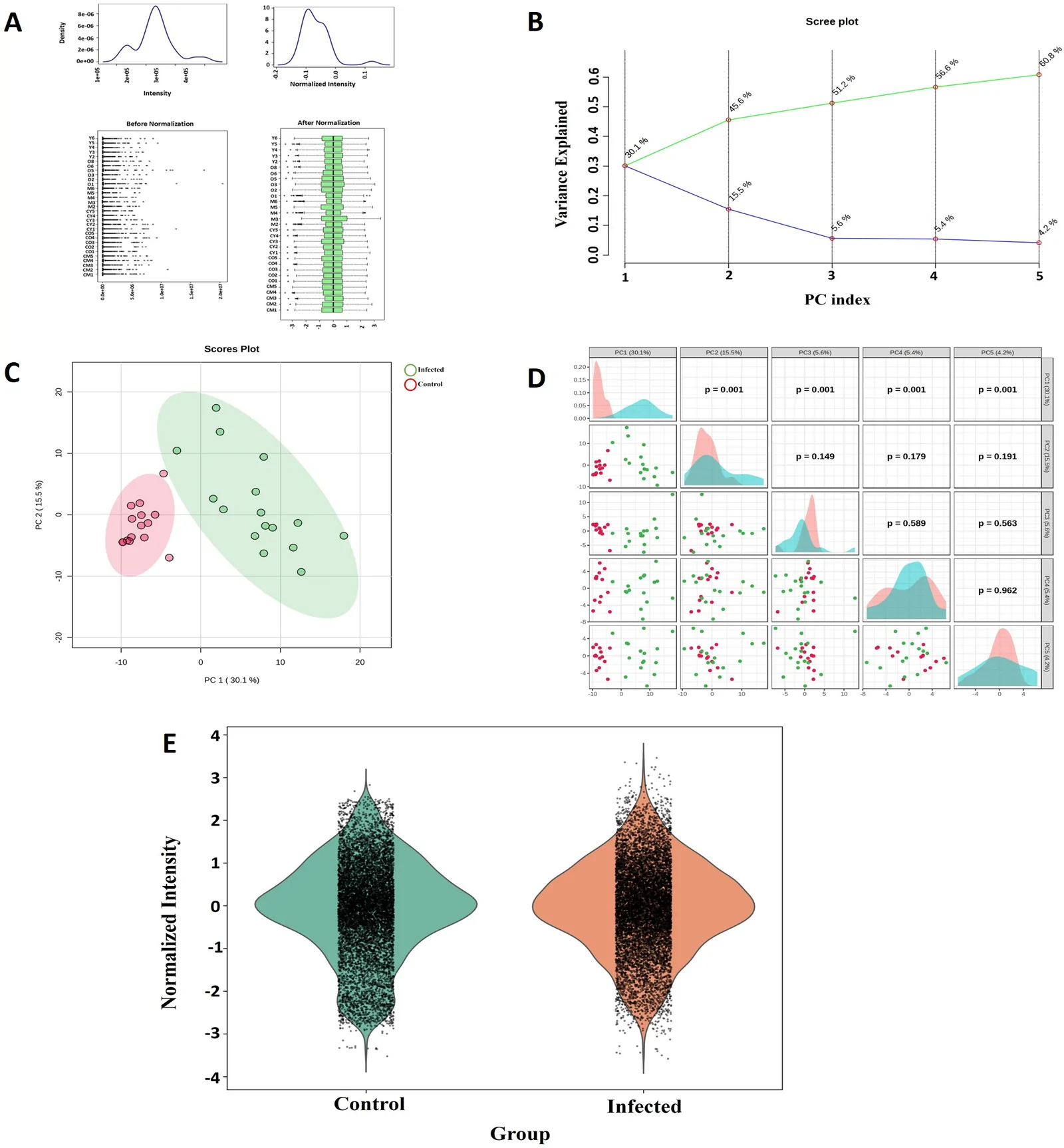
Bacterial endophthalmitis induces widespread metabolomic remodeling in human vitreous. (**A**) Density and box-and-whisker plots demonstrate improved symmetry and reduced inter-sample variability. (**B**) The scree plot highlights the proportion of variance. (**C**) PCA plots show distinct segregation between control and infectious vitreous along PC1 (30.1%) and PC2 (15.5%). (**D**) Pairwise principal component distributions further validate statistically significant group separation across multiple components. (**E**) Violin plots demonstrate significant alteration of metabolites in bacterial endophthalmitis.

Following normalization and log transformation, density-distribution plots illustrate that the metabolomic data showed near-Gaussian distributions with significantly reduced inter-sample variability. Consistently, box-plot analysis also showed minimal variability across samples (Fig. 1A). The first principal component (PC1) accounted for 30.1% of the total variance, and PC2 accounted for 15.5%, with the first five principal components accounting for 60.8% of the overall variance (Fig. 1B). Additionally, principal component analysis (PCA) plots demonstrated a clear segregation between vitreous samples of patients with bacterial endophthalmitis and non-infectious controls, which highlighted the pronounced infection-associated metabolomic reprogramming in the intraocular environment (Fig. 1C). Statistically significant differences between infected and control groups were observed across multiple components, where PC1 through PC5 showed significant separation (*P* = 0.001 for several component comparisons; Fig. 1D). Violin plot showed the widespread alterations in the metabolic shifts during infection (Fig. 1E). Overall, this demonstrates the robustness of the metabolic reprogramming during bacterial endophthalmitis in the vitreous.

### A non-linear dimensionality reduction reveals robust separation between infected and control vitreous metabolomes

**Fig 2:**
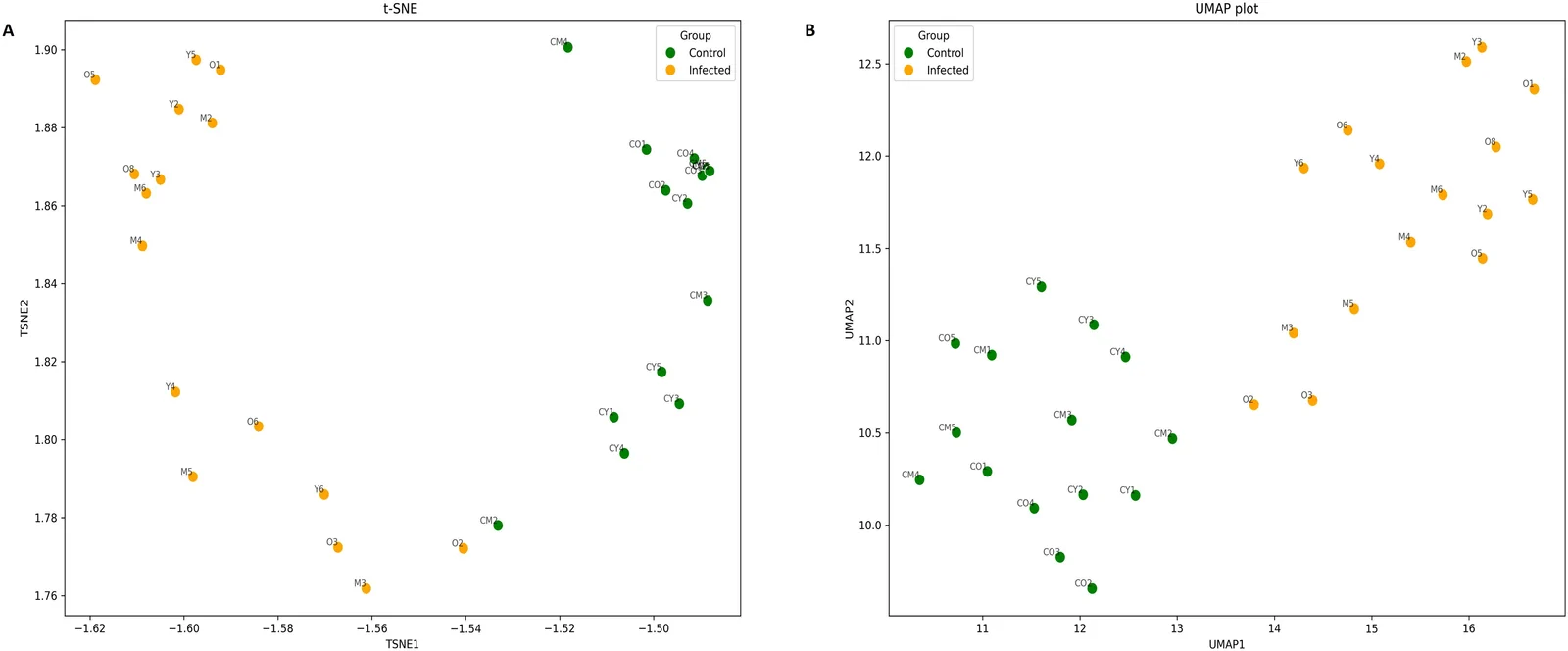
Nonlinear dimensionality reduction demonstrates clear metabolic separation between control and bacterial endophthalmitis vitreous samples. (**A**) The *t*-SNE plot shows distinct clustering of control and infected samples, with each point representing an individual vitreous sample. (**B**) UMAP analysis further confirms this separation, revealing consistent group-specific clustering and robust discrimination of infected samples from non-infectious controls in low-dimensional space.

To further assess group differences, non-linear dimensionality reduction was performed on the normalized metabolomics data. *t*-Distributed stochastic neighbor embedding (*t*-SNE) indicated the distinct clustering between the controls and infectious vitreous (Fig. 2A). Similarly, uniform manifold approximation and projection (UMAP) analysis also revealed robust discrimination between infected and control samples (Fig. 2B). This separation observed across both non-linear approaches highlights the presence of strong, reproducible metabolomic differences between bacterial endophthalmitis and non-infectious vitreous samples.

### Metabolomic profiling reveals infection-driven immune and oxidative metabolic remodeling in bacterial endophthalmitis

**Fig 3:**
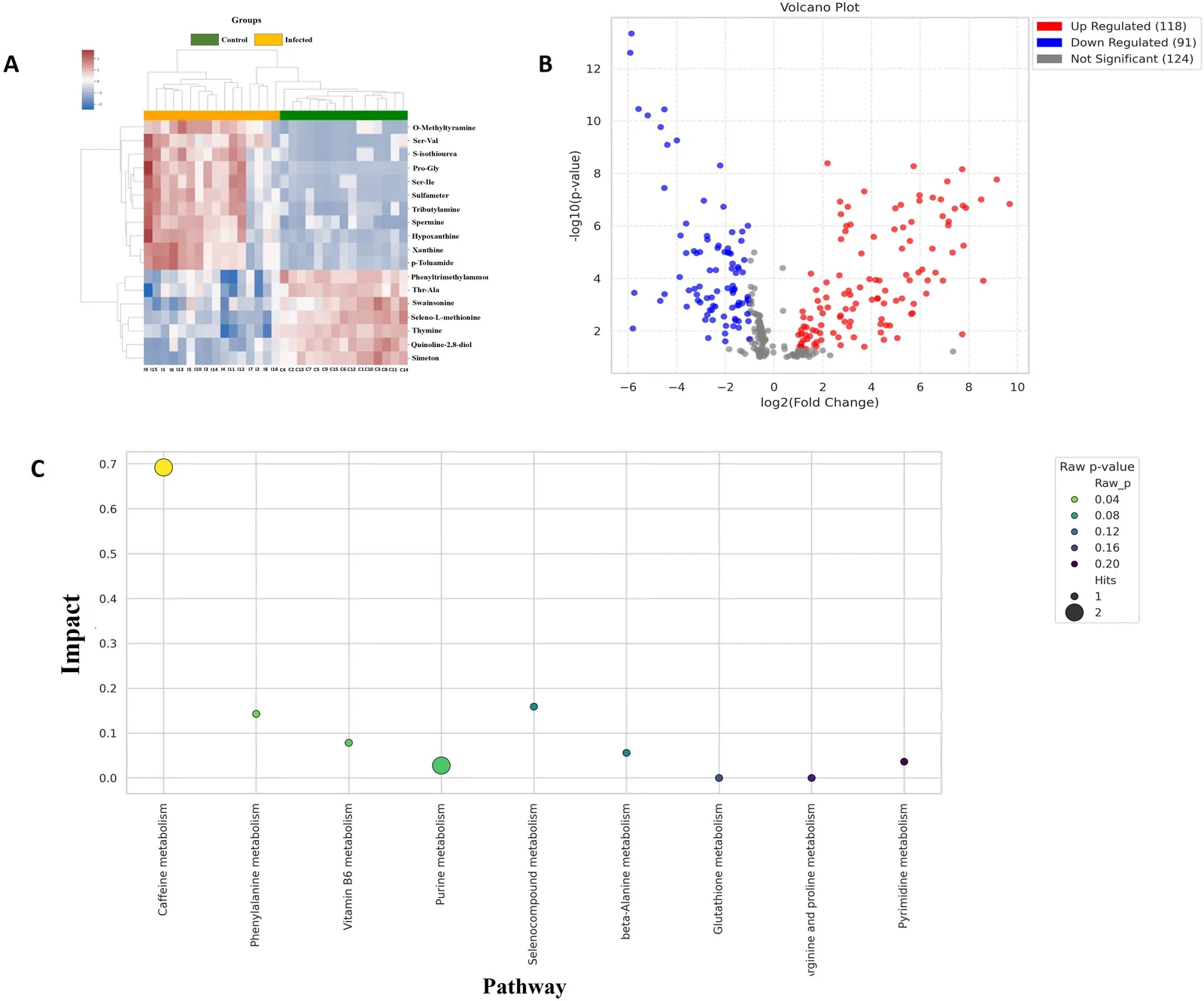
Differential metabolite abundance and pathway perturbations in bacterial endophthalmitis. (**A**) Heatmap of significantly altered metabolites demonstrates clear segregation between bacterial endophthalmitis and non-infectious control vitreous samples. (**B**) A volcano plot demonstrates differential metabolite abundance between infected and control groups. Significantly upregulated metabolites (*n* = 118) are shown in red, while significantly downregulated metabolites (*n* = 91) are shown in blue (*P* < 0.05), and non-significant features are indicated in gray. (**C**) Pathway impact analysis identifies multiple metabolic pathways significantly perturbed in infected vitreous, including purine metabolism, phenylalanine metabolism, vitamin B6 metabolism, selenocompound metabolism, beta-alanine metabolism, glutathione metabolism, arginine–proline metabolism, and pyrimidine metabolism.

Untargeted metabolomic profiling of human vitreous humor revealed pronounced metabolic distinctions between bacterial endophthalmitis samples and non-infectious controls. This segregation was driven by significant alterations in metabolites associated with amino acid metabolism, nucleotide turnover, polyamine synthesis, redox homeostasis, and xenobiotic pathways (Fig. 3A). The unique metabolic profile during infection indicates that the vitreous milieu is molded by intensified immune activation, oxidative stress, and tissue remodeling in the inflamed intraocular compartment. Among the most significantly elevated metabolites identified in infected vitreous humor were hypoxanthine and xanthine, key intermediates of purine catabolism (Fig. 3B). Accumulation of these metabolites reflects accelerated nucleotide breakdown and heightened xanthine oxidoreductase activity, both of which are hallmarks of oxidative stress and inflammatory signaling. Elevated purine metabolite levels are consistent with cellular damage, immune cell infiltration, and increased metabolic demand during bacterial infection. Polyamine metabolites, such as spermine, which modulates immune cell activation, oxidative stress, and microbial viability, were also significantly increased. It reflects the intertwined host immune responses and pathogen-driven metabolic interplay in the vitreous. Several dipeptides and amino acid conjugates, including seryl-valine, seryl-isoleucine, and prolylglycine, were likewise elevated in infected samples, suggesting enhanced proteolytic activity and altered amino acid turnover in the context of infection. During infection, these metabolic alterations are consistent with increased inflammatory protease activity and tissue degradation, culminating in peptide fragment release and accumulation within the vitreous. Additionally, elevated levels of S-methylthiourea and O-methyltyramine indicate perturbation in sulfur amino acid metabolism and catecholamine-associated pathways, processes that are commonly engaged during cellular stress and inflammatory responses. Infected vitreous metabolites that are associated with antioxidant defense were significantly reduced. Additionally, downregulation of metabolites of the glutathione pathway and seleno-L-methionine, which contributes to redox regulation, suggests that the antioxidant capacity is impaired and that there is elevated oxidative stress during bacterial endophthalmitis (Fig. 3C). Conversely, metabolites involved in the synthesis and degradation of nucleotides, such as thymine and quinoline 2,8-diol, were elevated.

The dysregulation of phenyltrimethylammonium cation and theanine reveals altered nitrogen metabolism and alterations in the amino acid-derived neuromodulatory pathways within the vitreous. In the context of bacterial endophthalmitis, pathway analysis demonstrated a significant perturbation in the metabolism of purines, phenylalanine, vitamin B6, selenocompounds, and beta-alanine. Vitamin B6-related pathways are essential for amino acid conversion reactions and appropriate immune cell function, while beta-alanine metabolism is associated with energy balance and cellular stress adaptation. Alterations in the metabolism of arginine and proline further influence the metabolic landscape of the infected vitreous, as evidenced by changes in nitric oxide signaling, extracellular matrix remodeling, and immune regulation.

Pathway analysis highlighted purine metabolism, phenylalanine metabolism, vitamin B6 metabolism, selenocompound metabolism, and beta-alanine metabolism as significantly dysregulated during bacterial endophthalmitis. Vitamin B6-dependent pathways are critical for amino acid interconversion and immune cell function, while beta-alanine metabolism is linked to cellular stress responses and energy homeostasis. Involvement of arginine and proline metabolism further implicates nitric oxide signaling, collagen turnover, and immune modulation in shaping the infected vitreous milieu.

Collectively, these metabolites and pathway-level alterations reveal that bacterial endophthalmitis involves coordinated disturbances in energy metabolism, amino acid turnover, nucleotide degradation, and redox homeostasis. The endurance of these biochemical signatures within the vitreous highlights their potential utility as indicators of infection-associated host responses, particularly in contexts where conventional microbiological cultures yield negative results.

### Lipidomic profiling of the vitreous humor in bacterial endophthalmitis reveals global infection-associated lipid remodeling

**Fig 4:**
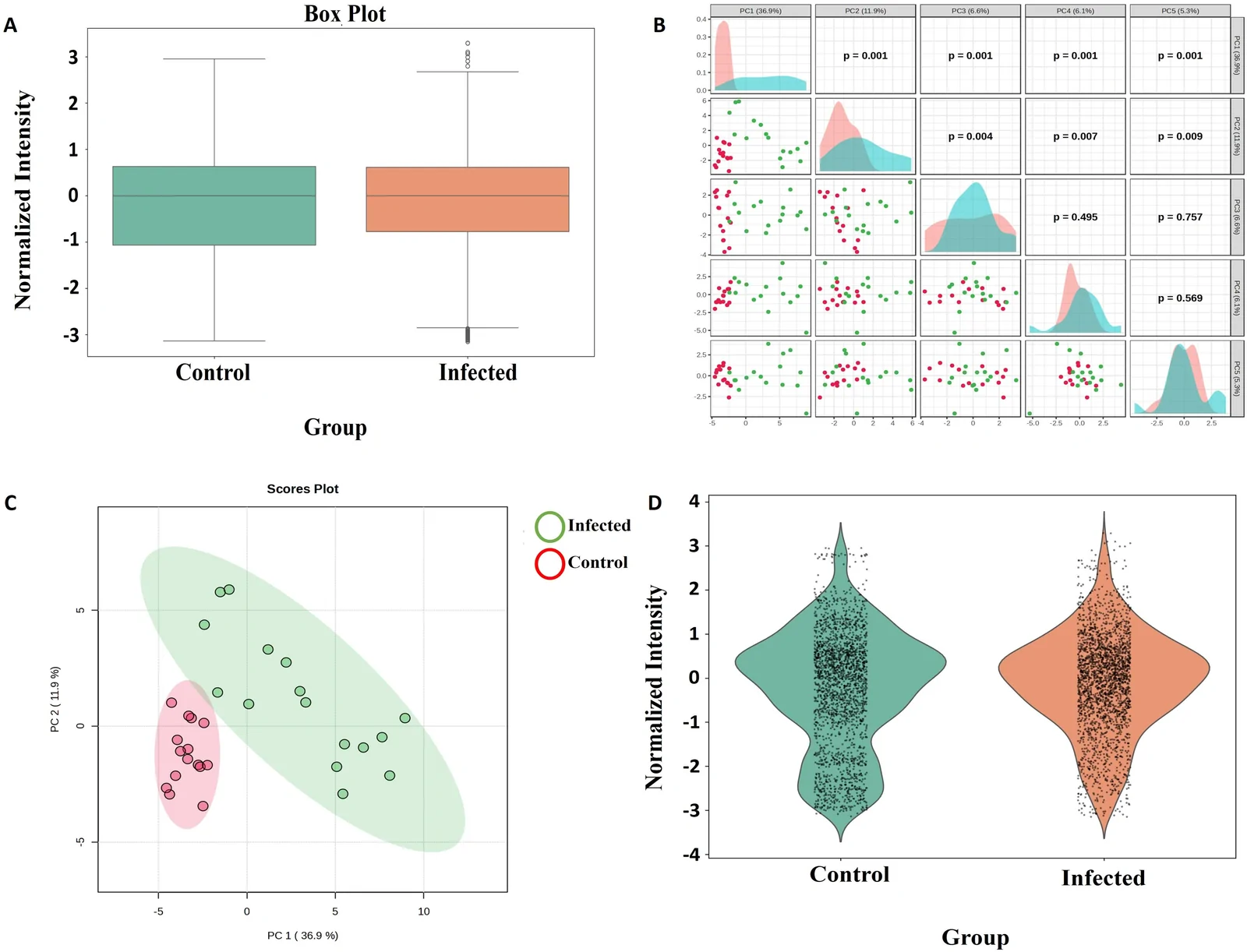
Comprehensive lipidomic reprogramming of the human vitreous in bacterial endophthalmitis. (**A**) Box-and-whisker plots depict normalized lipid intensity distributions in control versus bacterial endophthalmitis vitreous, confirming successful normalization and equivalent global signal ranges between groups. (**B**) Pairwise principal components density and correlation analysis of lipidomic data revealed marked separation between control and infected samples across the major principal components, as supported by permutation-based *P* values. (**C**) PCA score plots reveal clear clustering of bacterial endophthalmitis and control samples along PC1 (36.9%) and PC2 (11.9%), supporting infection-associated lipid remodeling. (**D**) Violin plots illustrate broad shifts in lipid abundance and signaling pathways in bacterial endophthalmitis.

We performed untargeted lipidomic profiling to characterize infection-associated biochemical alterations within the vitreous and to complement the metabolomic findings. Following normalization, comparable distributions were observed between control and infected samples (Fig. 4A). Multivariate analysis revealed clear segregation between vitreous samples from patients with bacterial endophthalmitis and non-infectious controls. PCA revealed clear separation of infected and control groups, reflecting profound lipidomics remodeling in intraocular infection (Fig. 4C). PC1 captured 36.9% of total variance, with PC2 accounting for 11.9%, together explaining much of the intergroup lipid heterogeneity. A statistically significant separation was observed in the group after pairwise principal component comparison (Fig. 4B), which supports the robustness of lipid-based classification. Violin plots show distinct shifts in normalized lipid intensity distributions between infected and control samples. All these findings indicate substantial remodeling of the vitreous lipid landscape, consistent with metabolic alteration.

### Differential lipid signatures and pathway dysregulation in bacterial endophthalmitis

**Fig 5:**
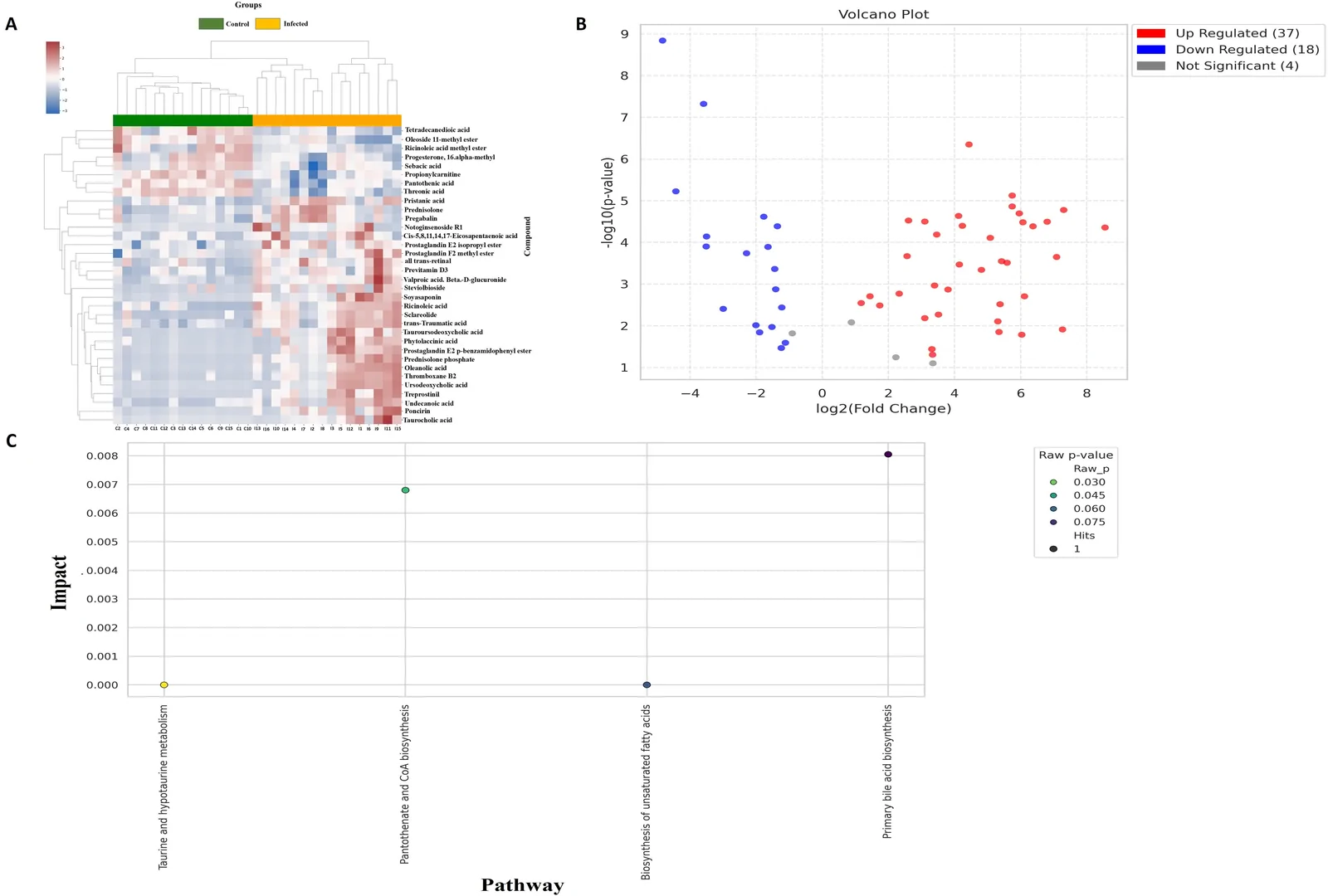
Differential lipid signatures and pathway perturbations in bacterial endophthalmitis. (**A**) Hierarchical clustering heatmap of significantly altered lipid species in vitreous samples from non-infectious controls and patients with bacterial endophthalmitis. (**B**) Volcano plot depicting differentially abundant lipids between infected and control vitreous samples. (**C**) Pathway enrichment analysis of altered lipids identifies primary bile acid biosynthesis, pantothenate and CoA biosynthesis, taurine and hypotaurine metabolism, and biosynthesis of unsaturated fatty acids as significantly affected pathways. Bubble size represents pathway impact, and color intensity corresponds to raw *P* values, emphasizing lipid-associated inflammatory and metabolic changes in bacterial endophthalmitis.

Hierarchical clustering of significantly altered lipids clearly revealed a distinct separation between bacterial endophthalmitis and non-infectious control vitreous, reflecting coordinated lipidomic shifts during intraocular infection (Fig. 5A). The heatmap showed persistent dysregulation across fatty acid metabolism, bile acid derivatives, eicosanoids, and membrane lipids, indicating a broad rewiring of lipid networks in infected eyes. Lipids associated with arachidonic acid metabolism, including PGE_2_, PGF, and related oxylipins, were markedly elevated. All these metabolites, known to promote inflammation and vascular permeability, align with the robust inflammatory response in endophthalmitis. Simultaneously, the increased abundance of unsaturated fatty acid-derived lipids indicates enhanced lipid peroxidation and activation of pro-inflammatory signaling pathways during infection. Substantial alterations were seen in bile acid-associated lipids, including tauroursodeoxycholic acid, taurocholic acid, and chenodeoxycholic acid-related compounds, which demonstrated clear grouping between the infected and control groups. Bile acid derivatives, once solely associated with hepatic metabolism, are now recognized for their roles in immunological regulation, mitochondrial stress responses, and cellular survival signals. Their presence in infected vitreous samples suggests a systemic-to-local metabolic connection and stress-responsive lipid signaling during severe intraocular infection. Furthermore, modifications in saturated and monounsaturated fatty acids, particularly derivatives of palmitic acid and stearic acid, were seen in bacterial endophthalmitis samples. These fatty acids are vital components of membrane remodeling and can influence innate immune activation, inflammasome signaling, and oxidative stress. The dysregulation of these lipid species is associated with membrane rupture, immune cell infiltration, and tissue destruction in the pathological vitreous environment. Univariate analysis, illustrated by volcano graphs, confirmed significant lipid dysregulation between infected and control samples (Fig. 5B). A total of 55 lipids demonstrated significant changes, with 37 lipids upregulated and 18 downregulated in bacterial endophthalmitis relative to controls (adjusted *P* < 0.05, log2 fold change >2), underscoring the magnitude and direction of infection-associated lipidomic variation. Pathway enrichment analysis of differentially regulated lipids identified several lipid-related metabolic pathways significantly disturbed during bacterial endophthalmitis (Fig. 5C). This included the metabolism of taurine and hypotaurine, the biosynthesis of pantothenate and CoA, the biosynthesis of unsaturated fatty acids, and the biosynthesis of primary bile acids. The disturbance of taurine and hypotaurine metabolism corresponds with the modulation of oxidative stress and cytoprotective mechanisms; yet, alterations in pantothenate and CoA production suggest increased lipid turnover and heightened energy demands during infection. The interruption of unsaturated fatty acid synthesis underscores the importance of lipid-mediated inflammatory signaling in the pathophysiology of bacterial endophthalmitis. The lipidomic alterations collectively suggest that bacterial endophthalmitis is coupled with the synchronized reorganization of inflammatory lipids, membrane-bound fatty acids, and bile acid-related metabolites. The lipidomic data, in conjunction with metabolomic discoveries, highlight infection-induced metabolic stress, increased inflammation, and membrane remodeling as fundamental biochemical traits of the infected vitreous environment.

### Validation of metabolites and lipid signature

**Fig 6:**
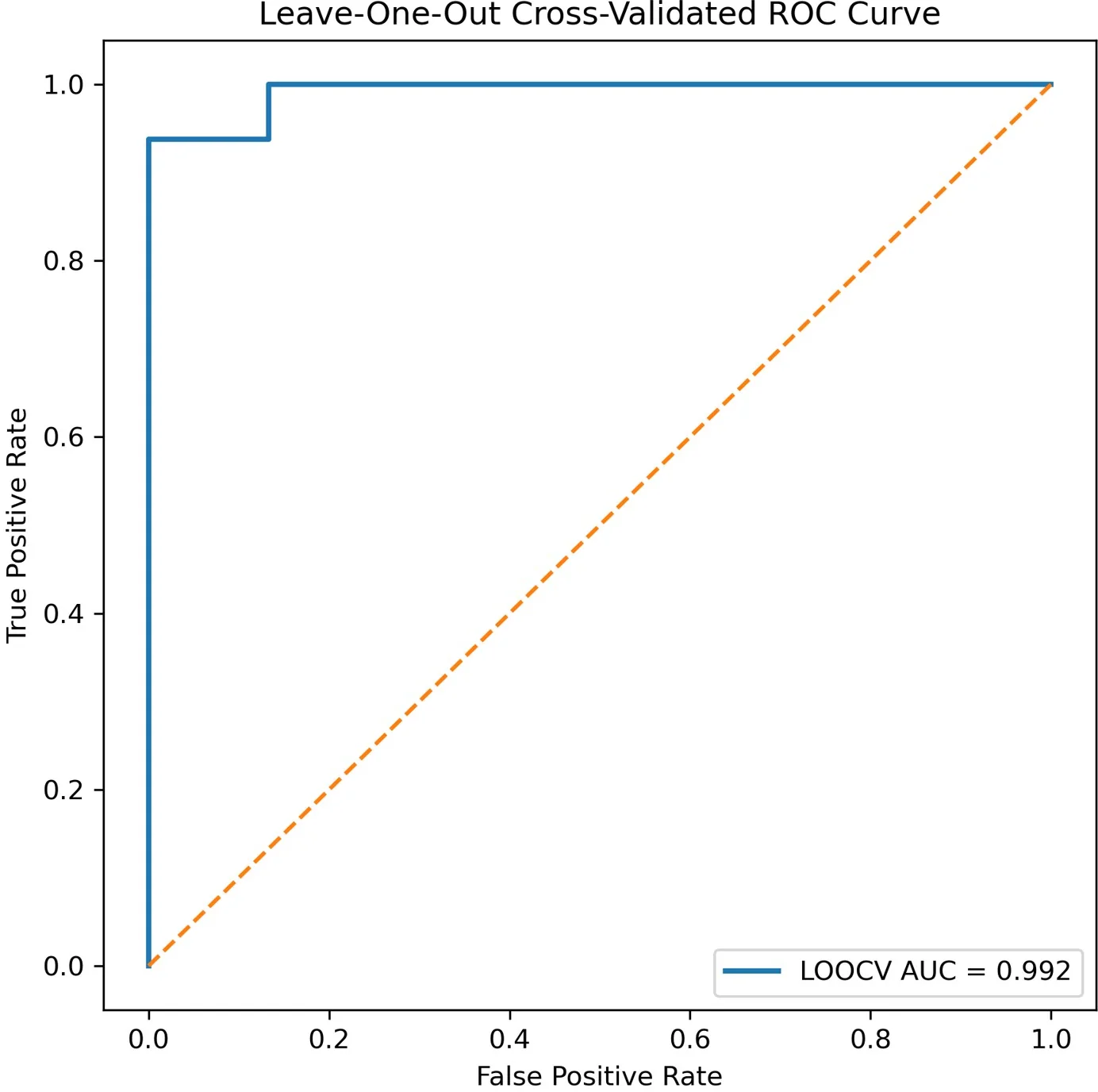
Receiver operating characteristic (ROC) curve generated using leave-one-out cross-validation (LOOCV) to evaluate the predictive performance of the identified metabolite/lipid signatures in distinguishing bacterial endophthalmitis (*n* = 16) from non-infectious controls (*n* = 15). The LOOCV-derived ROC curve (solid blue line) is plotted against the diagonal reference line representing chance-level discrimination (dashed orange). The aggregated predictions from all held-out samples yielded an area under the curve (AUC) of 0.992, with high sensitivity and specificity at a probability threshold of 0.5.

Predicted probabilities from all held-out samples were aggregated and used to construct receiver operating characteristic (ROC) curves and calculate classification metrics. The LOOCV analysis demonstrated excellent discriminatory performance, with an area under the ROC curve (AUC) of 0.992. Using a probability threshold of 0.5, the model also achieved high sensitivity and specificity for distinguishing infected cases from controls. These results indicate that the identified metabolite/lipid signature remains highly robust even under stringent cross-validation conditions designed to minimize overfitting. We have now included the LOOCV methodology, ROC analysis, and corresponding performance metrics in the revised Materials and Methods and Results sections (Fig. 6)

## DISCUSSION

Bacterial endophthalmitis represents an extreme inflammatory state within an otherwise immune-privileged ocular environment, characterized by rapid innate immune activation, profound metabolic stress, and irreversible retinal damage if left uncontrolled (22, 23). In this study, metabolomic and lipidomic analysis of human vitreous humor reveals coordinated remodeling of small-molecule metabolites and bioactive lipids in bacterial endophthalmitis, providing new insight into infection-driven biochemical reprogramming at both metabolic and lipid-signaling levels. This analysis offers new insights into the biochemical reprogramming of the body’s metabolism and lipid signaling that is influenced by infection. In both metabolomic and lipidomic data sets, unsupervised multivariate analysis consistently demonstrated a robust separation between infected and non-infectious control samples.

This suggests that bacterial infection imposes a dominant biochemical signature that surpasses inter-individual variability. The results of PCA, t-SNE, and UMAP exhibited a compact clustering of controls and a pronounced dispersion of infection samples. Infection severity, immune cell infiltration, and tissue injury are all associated with this dispersion, which is characterized by heterogeneous yet coordinated metabolic responses. It is important to emphasize that the observed group separation resulted from coordinated changes across multiple metabolic axes rather than by disruptions in individual pathways or isolated features. Such global metabolomic remodeling is particularly relevant in the context of endophthalmitis, where the restricted intraocular environment fosters the convergence of rapid bacterial growth, hypoxia, oxidative stress, and immune activation (24, 25). Dysregulation of purine metabolism, vitamin B6 metabolism, beta-alanine metabolism, selenocompound metabolism, arginine–proline metabolism, glutathione metabolism, and pyrimidine metabolism collectively indicates heightened nucleotide turnover, oxidative imbalance, and impaired redox homeostasis at the metabolite level. Elevated purine catabolites, such as hypoxanthine and xanthine, are indicative of accelerated ATP consumption, immune cell activation, and damage-associated nucleotide release. In addition, these metabolites are associated with elevated xanthine oxidoreductase activity, which enhances the generation of reactive oxygen species and inflammatory signaling (14, 16). Compromised antioxidant buffering is suggested by concurrent disruptions in glutathione and selenocompound metabolism, which exacerbate oxidative stress, a fundamental characteristic of infected vitreous. Inflammation, vascular dysfunction, and retinal tissue remodeling are all closely associated with nitric oxide signaling and collagen turnover, which are further implicated by changes in arginine–proline metabolism (26). Lipidomic profiling exposed the extensive remodeling of fatty acid-derived, eicosanoid, and bile acid-associated lipids, thereby substantiating the notion that lipids function not only as structural components but also as potent immunomodulatory mediators during bacterial endophthalmitis (27, 28). Of these, arachidonic acid-derived eicosanoids emerged as the dominant inflammatory lipid mediators of acute intraocular inflammation. Notably, prostaglandin E_2_ (PGE_2_), prostaglandin F_2_ɑ (PGF_2_ɑ), prostaglandin A_1_-associated derivatives, and thromboxane B_2_ (TXB_2_) were significantly elevated in infected vitreous samples (27–29). These lipid mediators are well-recognized amplifiers of inflammatory signaling and play a pivotal role in orchestrating intraocular immune responses. Functionally, prostaglandins and thromboxanes stimulate the production of key pro-inflammatory cytokines, including IL-1β, TNF-α, and IL-6, which collectively intensify the inflammatory cascade within the vitreous cavity. Their elevation contributes to increased vascular permeability and disruption of the blood-retinal barrier, facilitating the extravasation of immune cells into the normally immune-privileged ocular environment. Concurrently, eicosanoid signaling enhances the recruitment, activation, and survival of neutrophils, reinforcing the rapid bactericidal response while paradoxically contributing to collateral retinal tissue damage. Clinically, elevated levels of these lipid mediators are strongly associated with the development of pain, macular edema, retinal toxicity, and subsequent vision loss—hallmark features of bacterial endophthalmitis (16, 26). Taken together, these findings identify arachidonic acid-derived eicosanoids as key amplification circuits for acute inflammation, triggered by bacterial ingress and bridging tissue damage with host immune responses. Lipidomic analysis further revealed enhanced biosynthesis of unsaturated and free fatty acids, indicative of dynamic phospholipid membrane remodeling and increased phospholipase activity in response to bacterial toxins and immune-mediated signaling alongside augmented eicosanoid production (28). Beyond their role as biosynthetic precursors for prostaglandins and thromboxanes, free fatty acids themselves act as endogenous bioactive mediators capable of engaging innate immune receptors and intensifying inflammatory signaling responses. The lipid alterations identified are strongly consistent with metabolic evidence of increased energy demand, oxidative stress, and progressive tissue destruction, collectively underscoring the existence of a tightly coupled lipid-metabolite inflammatory axis. This axis appears to be functionally engaged during the pathogenesis of bacterial endophthalmitis. Interestingly, the metabolism of taurine–hypotaurine and bile acid-related lipids was also dysregulated (26, 30). Bile acids, which were traditionally associated with hepatic metabolism, are now more widely recognized as regulators of mitochondrial integrity, inflammasome activation, and immune cell survival through receptors like FXR and TGR5 (31). Notably, elevated arachidonic acid-derived eicosanoids, including PGE_2_, PGF_2_ɑ, and TXB_2_, identified in our study, have been previously linked to acute intraocular inflammation, vascular permeability, neutrophil recruitment, retinal toxicity, and vision loss during ocular infection and inflammatory retinal diseases (32, 33). Similarly, dysregulation of glutathione metabolism, taurine–hypotaurine metabolism, and purine metabolism observed in our data set is consistent with increased oxidative stress, mitochondrial dysfunction, retinal cell death, and tissue injury, all of which are recognized contributors to poor visual prognosis in endophthalmitis (34).

Importantly, the pronounced separation between infected and control samples observed across PCA, *t*-SNE, UMAP, and LOOCV analyses suggests that these metabolite–lipid signatures reflect biologically meaningful inflammatory and pathological states associated with infection severity. The observed dispersion among infected samples may further indicate heterogeneous host responses, immune infiltration, and varying degrees of retinal tissue damage.

**Fig 7:**
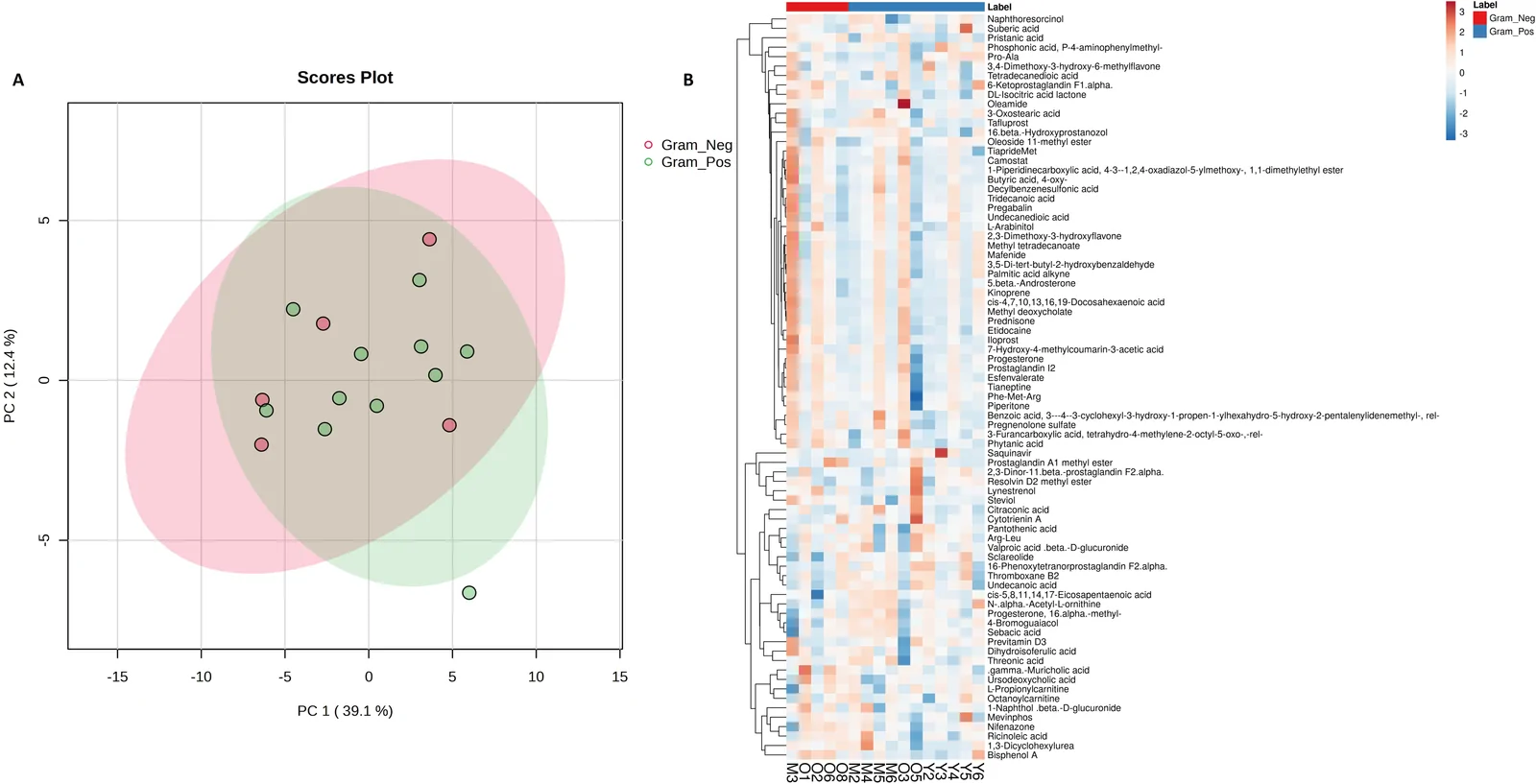
Multivariate analysis of metabolomic/lipidomic profiles in gram-positive and gram-negative bacterial endophthalmitis. (**A**) Principal component analysis (PCA) scores plot showing the distribution of gram-negative and gram-positive infected samples. (**B**) Heatmap with hierarchical clustering depicting the relative abundance of significant metabolites and lipids across individual samples.

We agree that correlating these signatures with clinical parameters such as visual acuity, retinal structural damage, inflammatory grading, and therapeutic response would substantially strengthen the translational significance of the study. Therefore, we have now included this point in the revised Discussion section as an important future direction. Larger longitudinal studies integrating metabolomic/lipidomic profiling with clinical outcome measures will be necessary to establish prognostic biomarkers and functional correlates in bacterial endophthalmitis. It is probable that their accumulation in the infected vitreous is the result of altered metabolic trafficking and stress-induced local lipid signaling during intraocular infection. The impairment of membrane stabilization and antioxidant defense is further implicated by the disruption of taurine and hypotaurine metabolism, which increases the susceptibility of retinal tissue to oxidative injury and lipid peroxidation (35). This study’s primary asset is the agreement between lipidomic and metabolomic findings. Pathways such as taurine–hypotaurine metabolism, pantothenate and CoA biosynthesis, and unsaturated fatty acid biosynthesis were identified as shared nodes that connect small-molecule metabolism and lipid turnover (36). The biosynthesis of pantothenate and CoA is a critical intersection of fatty acid synthesis, mitochondrial energetics, and immune cell metabolism, which highlights the increased metabolic flux that occurs during infection. The concurrent upregulation of inflammatory lipids and the depletion or redistribution of antioxidant and energy-regulating metabolites indicates a metabolic state that prioritizes immune defense over tissue homeostasis, which is likely responsible for the rapid retinal damage and poor visual outcomes that have been observed in clinical settings. Metabolite–lipid signatures identified in this study are intriguing candidates for disease stratification, prognosis, and therapeutic monitoring from a translational perspective. Our analysis did not reveal distinct clustering of metabolites between the bacterial groups, as reflected in both the heatmap and PCA plot. The PCA scores plot (Fig. 7) demonstrates two overlapping clustering patterns, with PC1 and PC2 accounting for 39.1% and 12.4% of the variance, respectively. The confidence ellipses for gram-negative and gram-positive cases overlap considerably, with most sample points falling within the shared central region, indicating that the two groups are not clearly segregated. However, certain metabolites and lipids, including suberic acid, oleamide, and 6-ketoprostaglandin F1-alpha, were consistently upregulated in gram-positive infected cases, as shown in the heatmap. In contrast, several other significant metabolites and lipids, such as naphthoresorcinol, tetradecanedioic acid, pristanic acid, and

butyric acid showed considerable overlap between the gram-positive and gram-negative groups. This heatmap pattern aligns with the PCA findings, further confirming the absence of clear segregation between gram-positive and gram-negative infected cases.

**Fig 8:**
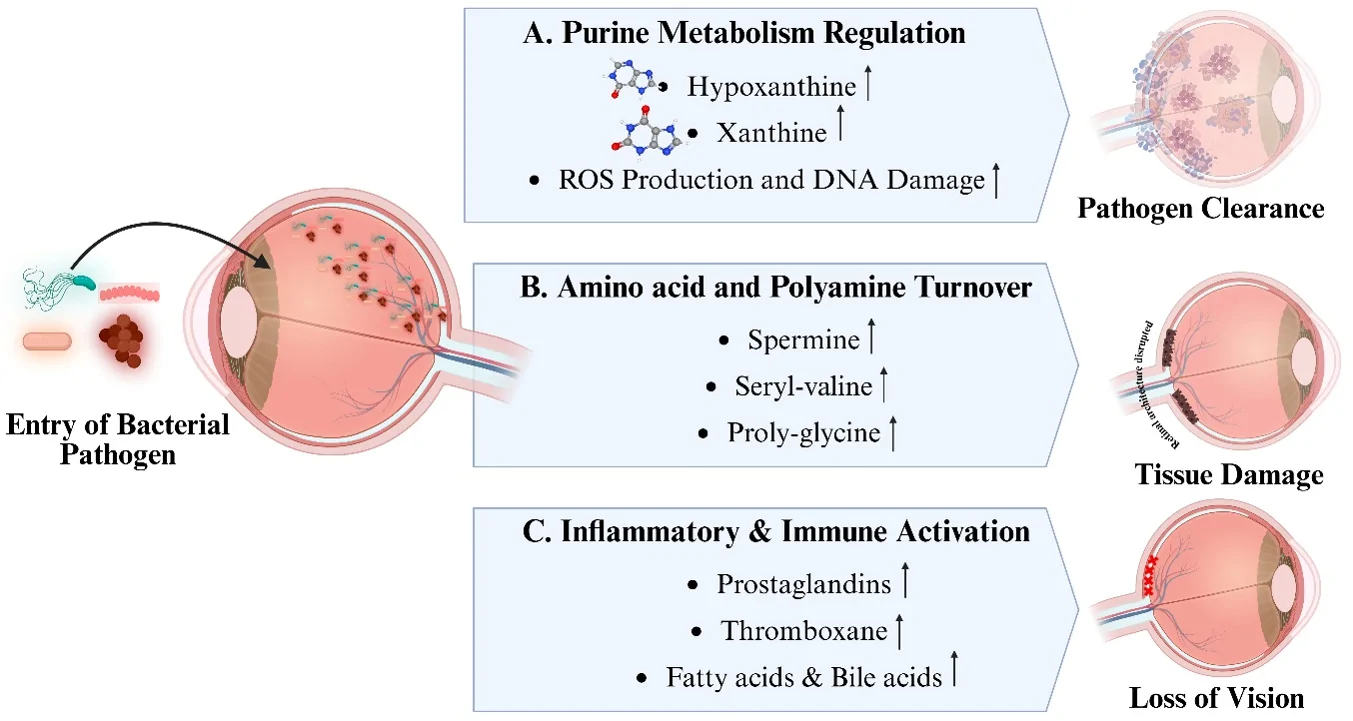
Summary diagram illustrating the key metabolic and lipid alterations associated with bacterial endophthalmitis.

Compared to individual inflammatory markers, metabolic and lipidomic profiles may provide a higher degree of sensitivity and specificity when separating infectious from non-infectious intraocular inflammation (Fig. 8). Additionally, this investigation has identified numerous pathways that are dysregulated and can be targeted with pharmacological agents. Particularly, the selective modulation of eicosanoid signaling has the potential to reduce destructive inflammation while simultaneously maintaining antimicrobial immunity (28). The stabilization of mitochondrial and CoA-dependent metabolism and the restoration of redox balance may serve as additional ancillaries to antimicrobial therapy, particularly in the context of severe or drug-resistant infections. A limitation of this study is the use of vitreous samples from patients with non-infectious retinal conditions as controls, which may introduce disease-related metabolic alterations. Furthermore, truly normal vitreous from healthy individuals is not clinically or ethically obtainable, while postmortem donor vitreous is subject to metabolic degradation and potential microbial contamination. Therefore, some observed differences may reflect both infection-specific and underlying disease-associated molecular changes. Overall, these results confirm that bacterial endophthalmitis is a condition that is exacerbated by host metabolic and lipidomic reprogramming, rather than solely by microbial burden. In ocular infections, the metabolite–lipid networks that have been identified here establish the groundwork for precision medicine approaches. Metabolic profiling has the potential to enhance visual outcomes, guide adjunctive therapy, and enlighten prognosis.

## Data Availability

The metabolomics data sets are publicly available through the Indian Metabolome Data Archive (IMDA) under the project/accession identifiers IMP_100076 / IMS_100076.

## AFTER EPUB

*[This article was published on 3 September 2026 with an incomplete Data Availability statement. The statement was corrected in the current version, posted on 24 September 2026.]*
